# Apresentação de Software para Pós-processamento de Curvas de Deformação Cardíaca: D-Station

**DOI:** 10.36660/abc.20180403

**Published:** 2020-04-06

**Authors:** Rafael Duarte de Sousa, Carlos Danilo Miranda Regis, Ittalo dos Santos Silva, Paulo Szewierenko, Renato de Aguiar Hortegal, Henry Abensur

**Affiliations:** 1 Instituto Federal de Educação, Ciência e Tecnologia da Paraíba João PessoaPB Brasil Instituto Federal de Educação, Ciência e Tecnologia da Paraíba,João Pessoa, PB – Brasil; 2 Instituto Dante Pazzanese de Cardiologia São PauloSP Brasil Instituto Dante Pazzanese de Cardiologia - Consultor Estatístico,São Paulo, SP – Brasil; 3 Instituto Dante Pazzanese de Cardiologia São PauloSP Brasil Instituto Dante Pazzanese de Cardiologia,São Paulo, SP – Brasil; 4 Hospital Beneficência Portuguesa de São Paulo Departamento de Ecocardiografia São PauloSP Brasil Hospital Beneficência Portuguesa de São Paulo – Departamento de Ecocardiografia, São Paulo, SP – Brasil

**Keywords:** Doenças Cardiovasculares/diagnóstico por imagem, Prognóstico, Ecocardiografia/métodos, Disfunção Ventricular Esquerda/fisiopatologia, Speckle Tracking

## Abstract

**Fundamento:**

O emprego de Speckle Tracking para estudo da função cardíaca tem grande aplicabilidade em diversos cenários. A expansão do uso deste método requer ferramentas que permitam a extração de dados relevantes das curvas de deformação cardíaca e que sejam adicionais aos parâmetros habitualmente utilizados.

**Objetivos:**

O presente trabalho visa apresentar e validar um software de uso livre, denominado D-station, para análise das curvas de deformação cardíaca.

**Métodos:**

A partir de arquivos raw data, o D-Station realiza a separação das fases do ciclo cardíaco, exibe simultaneamente curvas de Strain e Strain Rate de diferentes câmaras cardíacas. Para validação do software utilizamos o parâmetro Global Longitudinal Strain (GLS) avaliando-o: 1) Graficamente, a partir da comparação das Medidas emparelhadas de GLS no EchoPAC e D-Station frente à linha de igualdade; 2) pelo Coeficiente de Correlação dessas medidas; 3) pelo Teste de Hipóteses (p > 0,05); e 4) pelo Método Gráfico de Bland-Altman.

**Resultados:**

O Coeficiente rho de Spearman apontou forte correlação entre as medidas, o Teste de Hipóteses retornou um p-value = 0.6798 >> 0,05, que também indicou a equivalência entre elas; o Método gráfico de Bland-Altman mostrou um viés ≤ 1% e dispersão ≤ 2% entre as medidas. Os testes mostraram que para valores de GLS inferiores à 10% há a tendência de aumento das diferenças percentuais, mas seus valores absolutos ainda são baixos.

**Conclusão:**

O D-Station foi validado como uma aplicação suplementar ao EchoPAC que utiliza o raw data das curvas de Strain e Strain Rate obtidos por software proprietário. (Arq Bras Cardiol. 2020; 114(3):496-506)

## Introdução

A análise da deformação cardíaca pela técnica de *Speckle Tracking* tem grande aplicabilidade em diversos cenários, tanto de prática cardiológica ^1^ quanto no âmbito da pesquisa clínica, ^2^ provendo informações da mecânica regional e global das câmaras do coração.

O *Global Longitudinal Strain* (GLS) do ventrículo esquerdoé um parâmetro robusto de estudo da função cardíaca. ^1^ ^-^ ^3^ Contudo, este marcador avalia apenas a deformação que acontece entre o início do período de contração isovolumétrica e o final da fase de ejeção ventricular. Fases como o período de relaxamento isovolumétrico, por exemplo, podem conter informação valiosa não aferida pelo GLS.

Desta forma, se faz necessário o emprego de ferramentas que permitam a extração de dados relevantes das curvas de deformação cardíaca e que sejam adicionais aos parâmetros habitualmente utilizados.

A maioria dos softwares de processamento *offline* fornecidos pelos diferentes fabricantes ( *software* proprietário) tem um *setting* predeterminado de análises e parâmetros de deformação cardíaca. Isso, por um lado, torna o método mais simples e amigável para o uso na prática médica diária, mas, por outro lado, dificulta aplicações mais amplas dessa tecnologia na pesquisa clínica. Além disso, o acesso a essas ferramentas pode ser limitado e de alto custo.

É comum que centros internacionais de referência no estudo da deformação cardíaca tenham *softwares* customizados para a realização do processamento *offline* sem as limitações impostas pelos fabricantes, permitindo que sejam adaptados com base nas necessidades de suas pesquisas. ^4^

O presente trabalho visa demonstrar a aplicação de um novo *software* de uso livre, denominado D-station, como uma ferramenta para análise adicional das curvas de deformação cardíaca fornecidas por qualquer *software* proprietário *.* Além disso, ele pretende validar o novo software por meio da comparação de valores de GLS obtidos pelo D-Station com valores obtidos pelo software EchoPAC (GE).

## Métodos

### D-Station: software de Pós-Processamento das curvas de deformação cardíaca

**Figura 1:**
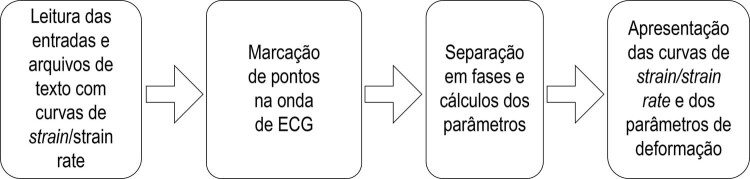
Algoritmo do D-station. ECG: eletrocardiograma.

O D-Station é um *software* customizado e gratuito, escrito em linguagem de programação *Python* 3 e direcionado para o pós-processamento *offline* das curvas de deformação cardíaca. Sua sequência de execução é apresentada na Figura 1 .

Ele não substitui as plataformas de processamento existentes, e sim atua como uma ferramenta que amplia as possibilidades pós-processamento.

### Divisão em fases

Cada curva de *strain* obtida corresponde a, no mínimo, um período do ciclo cardíaco em determinada região de uma câmara cardíaca e pode ser dividida nas fases mecânicas desse ciclo. Para isso, de acordo com estudos anteriores, ^4^ são necessários os tempos de abertura e de fechamento das valvas aórtica e mitral, e os tempos de eventos elétricos, obtidos a partir das ondas de eletrocardiograma (ECG), como o de início do primeiro e segundo complexo QRS, e de início da onda P. ^5^ ^,^ ^6^ As ondas de ECG acompanham as curvas de *strain* e *strain rate* (SR) nos arquivos.

Considerando o início de um ciclo cardíaco no início do complexo QRS, foram definidas seis fases. Em sua ordem de ocorrência, são elas: EMC - *Electrical Mechanical Coupling* (fase de acoplamento eletromecânico), IVC - *Isovolumic Contraction* (fase de contração isovolumétrica), Ejec - *Ejection Phase* (Fase de ejeção), IVR- *Isovolumic Relaxation* (fase de relaxamento isovolumétrico), E- *Early Filling* (fase de enchimento rápido), e A - *Atrial Contraction* (fase de contração atrial). Uma descrição detalhada da definição adotada para delimitar cada fase do ciclo cardíaco é realizada no material suplementar.

### Algoritmo de leitura dos sinais e cálculo de parâmetros

As entradas do programa são: 1) os tempos de abertura e fechamento das valvas aórtica e mitral; 2) os arquivos *raw data* contendo as curvas de *strain* ou SR; 3) o identificador do exame e 4) a opção de visualização selecionada pelo usuário. Mais informações podem ser encontradas nas instruções do programa, presentes no material suplementar do trabalho.

Na versão atual do *software* , são oferecidas ao usuário seis opções de visualização:

- *Strain - LV* ( *strain* do ventrículo esquerdo), *strain rate - LV* ( *SR* do ventrículo esquerdo) e ECG;
- *Strain - LV* , *strain - LA* ( *strain* do átrio esquerdo) e ECG;
- *Strain - LV* , *strain rate - LA* e ECG;
- *Strain - LV* , *strain - RV* ( *strain* do ventrículo direito) e ECG;
- *Strain - LV* , *strain rate - LV* e ECG, na qual o *SR* é obtido a partir das curvas de *strain* .
- *Opção de teste (para a interface CircAdapt): strain - LV* e *strain rate - LV*

**Figura 2:**
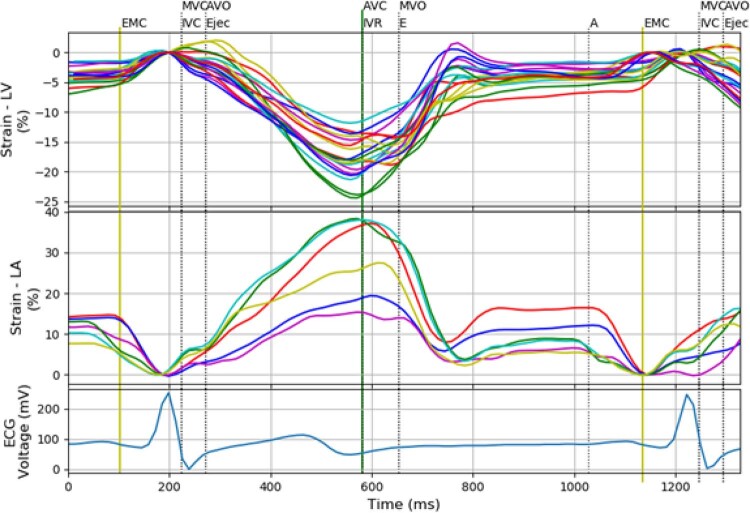
– Curvas de strain do ventrículo esquerdo, strain do átrio esquerdo, e do eletrocardiograma com a separação em fases. São exibidas 18 curvas de strain correspondentes aos 18 segmentos do ventrículo esquerdo, seis curvas de strain do átrio esquerdo e um sinal de eletrocardiograma. As cores nas curvas de strain e strain rate correspondem às cores atribuídas aos segmentos pelo software proprietário, sendo: MVC: Mitral Valve Closure; AVO: Aortic Valve Opening; AVC: Aortic Valve Closure; MVO: Mitral Valve Opening.

Em todas as opções, as curvas são exibidas simultaneamente, como apresentado na Figura 2 .

A partir dos arquivos de dados brutos ( *raw data* ) contendo a informação das janelas apical 3, 4 e 2 câmaras, são visualizadas as curvas de *strain* do ventrículo esquerdo, conforme o modelo de 18 segmentos proposto pela *American Heart Association* (AHA). ^7^

O processamento da planilha contendo os arquivos *raw data* consiste em alterar a formatação para facilitar o funcionamento do programa. Além disso, devido a pequenas diferenças na frequência cardíaca das curvas de ECG em cada arquivo, utilizamos como padrão o registro do corte 4 câmaras. Após a formatação, é exibida uma figura contendo curvas de *strain* , *SR* e o ECG. O usuário deve então marcar três pontos: o início do complexo QRS, o início da onda P e o início do segundo complexo QRS.

**Figura 3:**
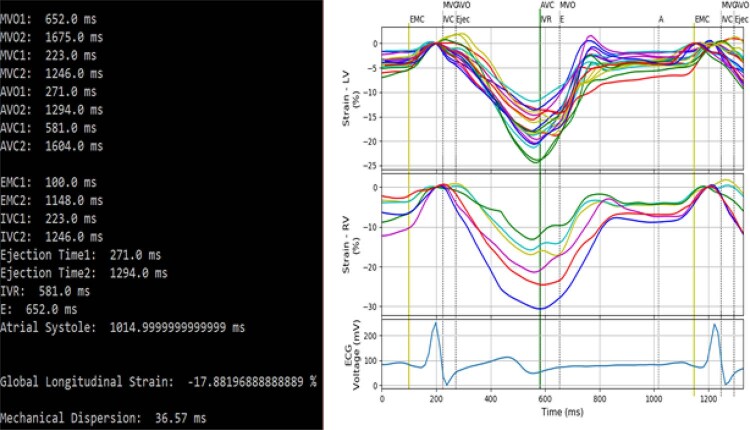
– À direita: visualização simultânea das curvas de strain longitudinal dos ventrículos esquerdo (18 segmentos) e direito (6 segmentos). À esquerda: os tempos de início das fases e os parâmetros calculados no terminal. Outras configurações também são possíveis de acordo com a opção escolhida, sendo MVC: Mitral Valve Closure, AVO: Aortic Valve Opening, AVC: Aortic Valve Closure, MVO: Mitral Valve Opening.

Com base nos valores de tempo obtidos a partir das marcações e nos tempos de abertura e fechamento das valvas, é possível determinar cada fase do ciclo cardíaco. No terminal do D-station, são exibidos os tempos de cada uma dessas fases, bem como os valores de cada parâmetro calculado. O usuário pode decidir entre uma figura que contém as curvas correspondentes às câmaras cardíacas de interesse (como exemplificada na Figura 3 ) ou uma figura com os pontos utilizados no cálculo de um determinado parâmetro.

### Marcação de eventos ( *event timing* ) e parâmetros calculados

Cada uma das curvas de *strain* longitudinal apresentadas na Figura 3 possui um ponto importante para o cálculo dos parâmetros do *software* : o *peak systolic strain* , definido, de acordo com o documento da EACVI/ASE, ^7^ como o ponto de maior contração durante a sístole.

O *peak systolic strain* de cada segmento é utilizado no cálculo do parâmetro GLS, definido como a média aritmética dos valores de *peak systolic strain* de todos os segmentos.

As possibilidades de pós-processamento permitem e/ou facilitam a análise de novos parâmetros como *strain* /SR AE e AD, *strain* VD e *diastolic recovery* ( *diastolic stunning* ) ^8^ por exemplo.

### Algoritmo de reconhecimento do *peak systolic strain*

**Figura 4:**
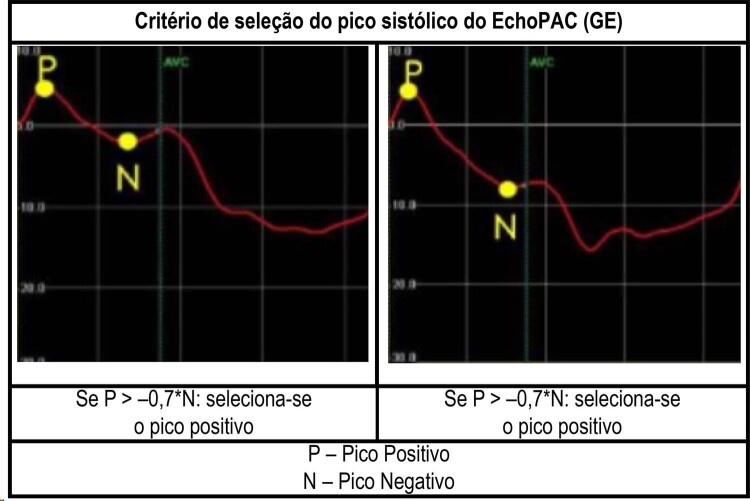
– Critério para seleção do pico sistólico utilizado pelo EchoPAC.

O D-Station considera o *peak systolic strain* como o valor mais negativo do conjunto de pontos existentes entre o início do QRS e o AVC. Isso diverge do critério do *software* EchoPAC que seleciona o *systolic peak strain* de acordo com a regra apresentada na Figura 4 :

### Validação do D-Station: banco de dados e análise estatística

Para validação do D-Station, arquivos com curvas de deformação de 48 indivíduos foram obtidos da base de dados do setor de Ecocardiografia do Hospital Beneficiência Portuguesa de São Paulo. Não houve cálculo de tamanho amostral, sendo usada uma amostra por conveniência ( *convenience sampling* ) por análise retrospectiva. Todos os exames foram realizados mediante assinatura do termo de consentimento. O estudo foi aprovado pelo comitê de ética desta instituição sob o número de protocolo CAEE 91350318.4.0000.5483. Utilizando o EchoPAC (versão 202 GE), foram inspecionados os tempos registrados de abertura e fechamento das valvas mitral e aórtica. Alguns exames tinham mais de um registro dos tempos de eventos; exames com discrepâncias de tempo maior que 10 ms foram excluídos.

A seguir, selecionou-se o ciclo cardíaco com melhor qualidade de imagem nas janelas apical 3, 4 e 2 câmaras. O último ciclo foi selecionado nos casos de três ciclos com qualidade satisfatória. A borda endocárdica foi tracejada delineando a região de interesse pela opção *Q-analysis* do programa EchoPAC. Foi feita então a inspeção visual de qualidade do “ *tracking* ” e, se pertinente, confirmada pela opção “ *approve* ”, seguido do registro do valor do GLS_EchoPAC. Em caso de *tracking* visualmente inadequado, o processo foi repetido. Exames com mais de dois segmentos inadequados foram excluídos.

O *raw data* das curvas de deformação foi exportado pela opção “ *Store Trace* ”, a qual gera arquivos .txt que são utilizados para o processamento no D-Station.

O GLS foi escolhido como parâmetro de validação da equivalência das medidas no processamento pelo EchoPAC (técnica já estabelecida – “gold standard”) e o D-Station (técnica alternativa proposta), apresentados na Tabela 1 .

**Tabela 1:** – Valores de Global Longitudinal Strain (GLS)(%) fornecidos pelo EchoPAC e pelo D-Station

Sujeito	GLS_Echopac	GLS-D-Station	Sujeito	GLS_Echopac	GLS-D-Station	Sujeito	GLS_Echopac	GLS-D-Station
1	–17,90	–17,88	17	–19,00	–19,03	33	–24,40	–24,62
2	–7,90	–9,50	18	–16,90	–16,82	34	–19,10	–19,57
3	–10,50	–11,10	19	–19,500	–16,68	35	–7,40	–6,46
4	–8,50	–8,19	20	–19,80	–19,83	36	–2,70	–3,37
5	–13,30	–13,55	21	–16,70	–17,04	37	–5,70	–5,22
6	–18,40	–18,26	22	–20,50	–20,93	38	–4,50	–4,32
7	–4,60	–4,21	23	–14,90	–14,71	39	–10,50	–9,83
8	–21,60	–21,48	24	–20,20	–19,76	40	–9,40	–10,95
9	–16,20	–16,36	25	–17,80	–18,19	41	–10,60	–10,47
10	–11,90	–11,41	26	–20,10	–20,47	42	–11,10	–11,15
11	–8,80	–7,33	27	–17,30	–17,60	43	–3,20	–3,69
12	–17,30	–17,23	28	–17,50	–16,96	44	–8,20	–8,64
13	–20,40	–20,32	29	–21,20	–20,28	45	–6,60	–6,01
14	–19,80	–19,40	30	– 23,00	–23,06	46	–6,90	–6,85
15	–16,40	–15,27	31	– 20,70	–19,91	47	–10,60	–10,11
16	–19,20	–19,38	32	–21,10	–21,22	48	–8,80	–9,28

### Métodos utilizados nas análises:

- Teste de normalidade das medidas de GLS do EchoPAC, D-Station e das diferenças EchoPAC - D-Station, utilizando-se um método gráfico (Gráfico Q-Q) seguido de um método estatístico (Teste de Shapiro-Wilk) para confirmação da ocorrência de normalidade pelo método gráfico;
- Gráfico das medidas de GLS do EchoPAC e D-Station frente à linha de igualdade e coeficiente de correlação (método de Pearson caso os dados do EchoPAC e D-Station apresentem normalidade ou método de Spearman, caso contrário);
- Teste de hipóteses das diferenças das medidas de GLS (EchoPAC - D-Station), dados pareados, nível de significância de 5%, utilizando-se o teste t de Student caso as medidas das diferenças apresentem normalidade ou o teste não Paramétrico de Wilcoxon, caso contrário.
- Análise de concordância pelo método de Bland-Altman ^9^ ^,^ ^10^

Foi utilizado o *software R version* 3.5.2 (2018-12-20) que, por meio dos pacotes Stats e BlandAltmanLeh, contempla os comandos necessários e *outputs* dos métodos de obtenção do p-value e Bland-Altman.

### Critérios de validação

Do ponto de vista clínico, consideramos como critérios para reconhecer o D-Station como alternativa ao EchoPAC (equivalência):

- Teste de normalidade A avaliação pelo gráfico Q-Q é visual e, portanto, subjetiva. O padrão da normalidade dos dados é o alinhamento dos pontos em uma linha reta traçada pelo algoritmo a partir dos dados avaliados. A aceitação da hipótese de normalidade pelo teste de Shapiro-Wilk se dá quando p-value > α (nível de significância = 5%).
- Coeficiente de correlação de Spearman ≥ 0.95
- Teste de hipótese: p-value > 0.05 (há equivalência entre as medidas) Ho: média das diferenças (EchoPAC - D-Station) = 0 Ha: média das diferenças ≠ 0
- Bland-Altman

- erro sistemático (“ *bias* ” ou viés) ≤ 1%
- dispersão ≤ 2%

(*) Notar que a unidade de medida do GLS é % e, portanto, os valores adotados dizem respeito à variação absoluta.

## Resultados

### Visualização simultânea de curvas de diferentes Câmaras Cardíacas

**Figura 5:**
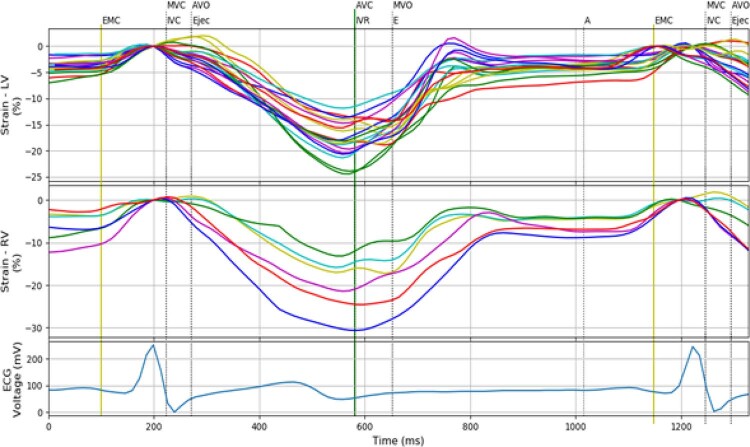
– Apresentação simultânea das 18 curvas de strain do ventrículo esquerdo, seis curvas de strain do ventrículo direito e curva de eletrocardiograma, sendo MVC: Mitral Valve Closure; AVO: Aortic Valve Opening; AVC: Aortic Valve Closure; MVO: Mitral Valve Opening.

O D-Station permite a opção de exibição simultânea de todas as curvas de *strain* e *SR* de diferentes câmaras cardíacas, permitindo o estudo da interação entre elas. Opções adicionais com combinações de diferentes exibições podem ser facilmente adicionadas, permitindo a extração de outros parâmetros para estudo da deformação em diferentes câmaras simultaneamente e em cada período do ciclo cardíaco. Como exemplo, na Figura 5 são exibidas as curvas do ventrículo esquerdo e direito, facilitando o estudo das interações interventriculares.

### Interface CircAdapt: geração de modelos cardíacos virtuais

**Figura 6:**
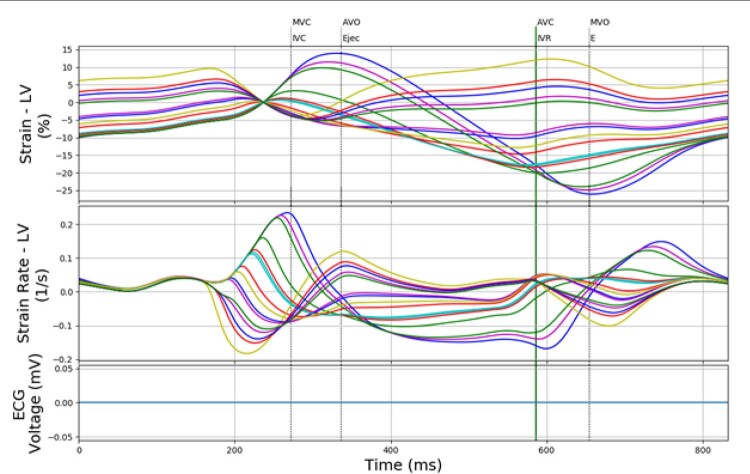
– Apresentação simultânea das 18 curvas de strain do ventrículo esquerdo e 18 curvas de strain rate do ventrículo esquerdo provenientes do CircAdapt; portanto, não há sinal de eletrocardiograma e a separação em fases realizada deve ser desconsiderada, sendo MVC: mitral valve closure; AVO: aortic valve opening; AVC: aortic valve closure; MVO: mitral valve opening.

A opção “Test” no *software* visa a determinação dos parâmetros das curvas de *strain* sem a separação de fases. Com isso, a curva de ECG não é mais necessária, e o programa torna-se compatível com o modelo matemático CircAdapt. Esse modelo associado ao MultiPatch Module, apresentado por Walmsley et al., ^11^ pode retornar curvas de strain correspondentes às simulações e os tempos de eventos mecânicos, sem sinais de ECG, como mostrado na Figura 6 . Dessa forma, o software desenvolvido pode trabalhar com modelos cardíacos virtuais elaborados seguindo o trabalho de Walmsley et al. ^11^ ^-^ ^14^

### Possibilidade de aplicação de técnicas de aprendizado de máquina ( *Machine Learning* )

*Machine learning* consiste em uma área do campo da inteligência artificial capaz de processar problemas complexos de interação entre variáveis e fazer predições acuradas. Tem sido amplamente utilizada em diferentes áreas da cardiologia.

O formato de armazenamento das entradas e dados obtidos pelo *software* permite implementar algoritmos de *machine learning* , os quais possibilitam a extração automática de parâmetros, classificação de grande número de sinais e leitura das características espaço-tempo de toda a curva de deformação como proposto por Tabassian et al. ^15^

### Resultados da análise de validação

#### a) Teste de normalidade das medidas

**Figura 7:**
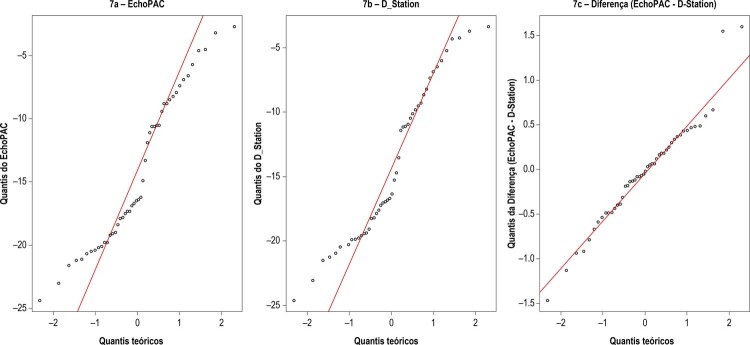
– Gráficos Q-Q.

A Figura 7 apresenta os gráficos Q-Q das medidas do EchoPAC ( Figura 7a ), D_Station ( Figura 7b ) e da diferença EchoPAC - D-Station ( Figura 7c ). Observa-se nas Figuras 7a e 7b , que muitos pontos estão fora da linha reta de referência traçada em vermelho, indicando que as medidas do EchoPAC e do D-Station não são normalmente distribuídas. Já a Figura 7c apresenta a maioria dos pontos sobre ou bem próximos da linha reta de referência traçada em vermelho (exceto dois pontos no extremo superior direito do gráfico), indicando que as diferenças entre as medidas tendem a se distribuir normalmente.

**Figura 8:**
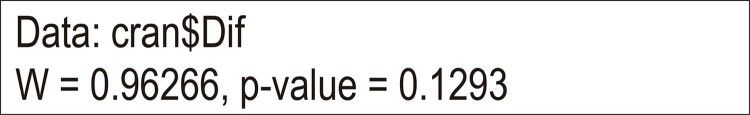
– Shapiro-Wilk normality test.

Como as diferenças das medidas serão utilizadas no teste de hipóteses, buscamos confirmar a hipótese de normalidade na distribuição dessas diferenças obtidas pelo método gráfico, aplicando o teste de Shapiro-Wilk, cujo resultado apresentado na Figura 8 confirma a hipótese de normalidade (p > 0,05).

#### b) Gráfico das medidas do EchoPAC e D-Station frente à linha de igualdade e coeficiente de correlação

**Figura 9:**
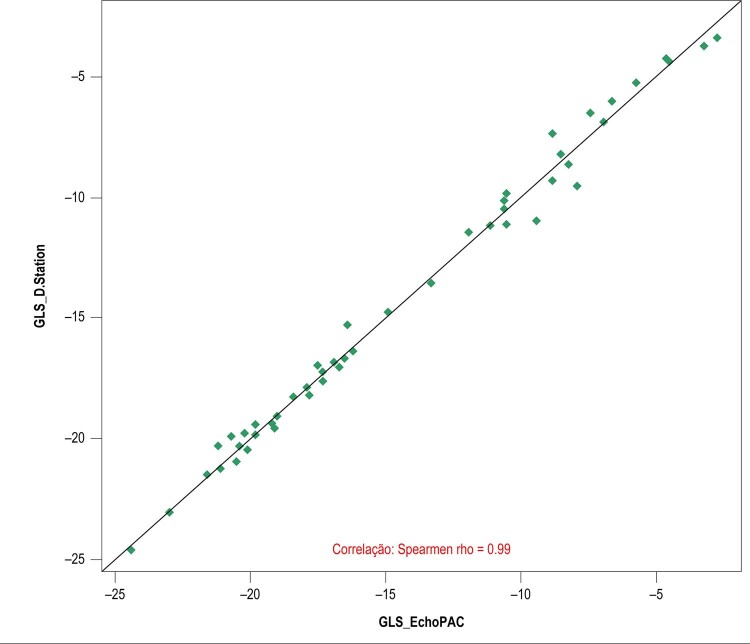
– Valores de Global Longitudinal Strain (GLS) fornecidos pelo EchoPAC e pelo D-Station com a linha de igualdade.

A Figura 9 apresenta a distribuição dos valores do EchoPAC e D-Station (dados pareados) frente a linha da igualdade, mostrando uma distribuição dos pontos próximos e em ambos os lados dessa linha, apontando, do ponto de vista qualitativo, para um baixo viés ( *bias* ) e dispersão. Como essas medidas não são normalmente distribuídas, foi calculado o coeficiente de correlação de Spearman, que indicou uma forte correlação (r = 0,99) entre as medidas dos dois métodos.

#### c) Teste de hipóteses das diferenças das medidas de GLS (EchoPAC - D-Station)

**Figura 10:**
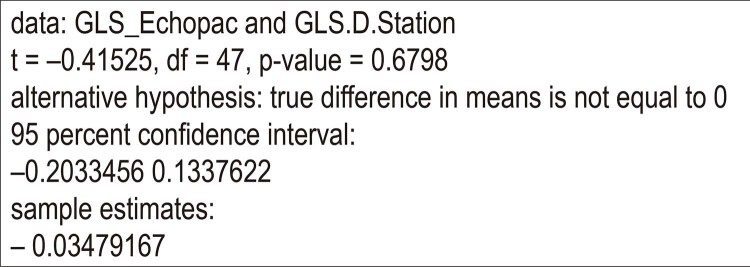
– Paired t-test.

Como as diferenças entre as medidas apresenta distribuição normal, aplicamos o teste t de Student para dados pareados, e nível de significância de 5%, com os resultados apresentados na Figura 10 . Obtivemos um valor p de 0,6798, apontando para a aceitação da hipótese nula (ou seja, equivalência dos métodos).

#### d) Análise de concordância entre métodos de Bland-Altman9,10

**Figura 11:**
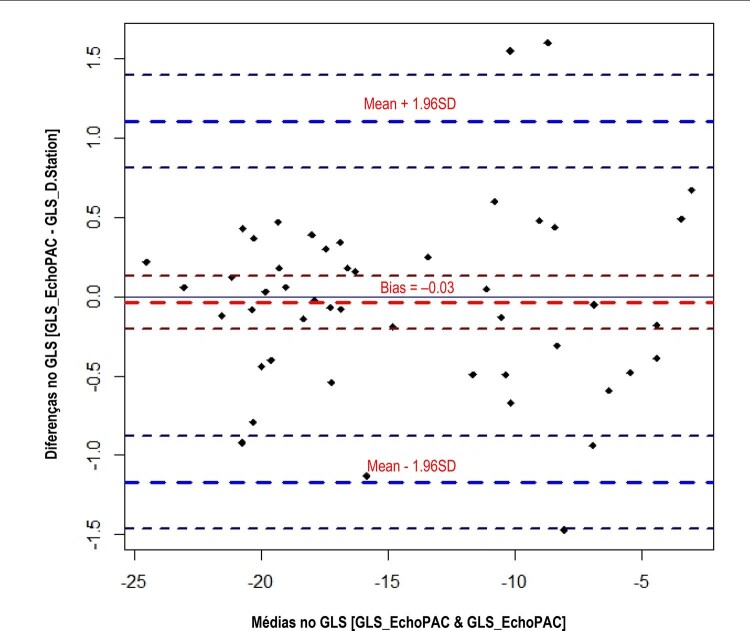
– Diferenças e médias dos valores de global longitudinal strain (GLS) obtidos pelo EchoPAC e pelo D-station.

A Figura 11 mostra o gráfico de Bland-Altman, que indica a concordância entre os dois métodos ao atender o item c) dos critérios de validação. Há uma indicação de diferenças % maiores para valores absoluto (módulo) de GLS < 10%.

## Discussão

### Análise de concordância entre métodos

Por atenderem os critérios de validação, os resultados da análise de validação indicam equivalência entre medições de GLS do EchoPAC e D-Station. Em uma análise detalhada dos dados, observa-se que, para medidas inferiores a 10%, houve tendência para diferenças percentuais maiores. Curiosamente, todos esses sujeitos eram portadores de disfunção ventricular importante com dissincronia tipo bloqueio de ramo esquerdo. Alguns fatores podem precipitar as discrepâncias apontadas:

1. Valores absolutos baixos resultam em diferenças percentuais maiores;
2. O padrão de dissincronia tipo bloqueio de ramo esquerdo costuma apresentar o " *stretching* " do segmento basal da parede inferolateral e/ou anterolateral no início da sístole, bem como movimentos erráticos médio e telessistólicos do septo após o característico " *septal flash* ". Ambos podem resultar no surgimento de picos positivos. Enquanto o D-Station considera como o *systolic peak* o valor mais negativo, independentemente do pico positivo ou menos positivo em caso de curvas exclusivamente positivas, o EchoPAC utiliza como regra de pico sistólico ( *peak systolic strain* ), o pico positivo quando este supera 75% do valor modular do pico sistólico negativo, como detalhado anteriormente na Figura 4 . Ademais, no EchoPAC, é comum utilizar ajustes manuais nesses casos, porém optamos por não incluir estes ajustes visando maior rigor metodológico.

Em suma, a divergência na definição do *systolic peak* reduz a reprodutibilidade do GLS entre os softwares nos pacientes com dissincronia tipo bloqueio de ramo esquerdo. Essa questão deve ser abordada em estudos subsequentes.

Tais discrepâncias, contudo, não têm impacto significativo, especialmente quando consideramos a variabilidade intraexaminador do GLS reportada na literatura (estimada em 5,2%), ^16^ bem como as divergências entre softwares pertinentes aos algoritmos de filtragem ( *speckle filtering* *and* *tracking* ). ^17^ ^-^ ^19^

Assim, as análises dos dados apresentados validam o D-Station como alternativa ao EchoPAC.

### Potenciais aplicações do software D-Station

As possibilidades de utilização do software são diversas: a avaliação simultânea de diferentes câmaras permite estudar a interação entre as deformações entre ventrículos esquerdo e direito, e entre ventrículo esquerdo e átrio esquerdo, o que pode ser relevante nos contextos de insuficiência cardíaca com fração de ejeção preservada, pericardiopatias e dissincronia interventricular.

A interface do D-Station com o modelo Circadapt associado ao MultiPatch Module permite formulação de hipóteses e comparação com sinais de pacientes reais como já realizado previamente. ^12^ ^-^ ^14^ Isso gera recursos de ensino da fisiopatologia da deformação cardíaca, além de potencialmente reduzir o tempo da escolha das variáveis de interesse e poupar recursos com elaboração de modelos animais em alguns cenários de pesquisa.

A aplicação de *Machine Learning* pode ser configurada para processamento de uma grande quantidade de sinais, identificar variáveis de interesse por meio da mineração de dados ( *data mining* ), além de permitir o uso de todos os pontos da curva de *strain* /SR como o descrito por Tabassian et al. ^15^ Esse raciocínio pode ter desdobramentos em como extrair mais dados relevantes a partir do estudo deformação cardíaca potencializada pelas técnicas de *machine learning* , sobretudo com a chegada iminente do " *High Frame Rate Speckle Tracking.* ^*20*^ *"*

Por fim, atualizações futuras devem expandir as opções para análise com *Strain* Radial, Circunferencial e *Twist* , e otimizar a interface entre os *softwares* proprietários incorporando aos parâmetros de deformação, sinais extraídos de Doppler, volumes das câmaras, *Tissue Doppler* , dentre outros. Isto permitirá a extração automatizada de uma nova série de parâmetros predeterminados a critério do usuário.

### Limitações

A versão atual do *software* D-Station não permite que as visualizações sejam atualizadas. Isto é, para alterar a escolha das câmaras que terão suas curvas de Strain/SR exibidas, o usuário deve reiniciar o programa. O mesmo acontece caso haja marcação errada no sinal de ECG.

A diferença de aferição do *strain* cardíaco entre diferentes fabricantes é uma questão crítica a técnica de speckle tracking, com o problema já discutido adequadamente por Mirea et al. ^18^ Estudos adicionais são necessários para avaliar o impacto deste software nas divergências de resultados entre fabricantes.

## Conclusão

O *software* D-station é uma ferramenta adicional de avaliação das curvas de deformação cardíaca obtidas por meio do *raw data* exportado de outro *software* proprietário, com boa correlação da aferição do GLS quando comparada ao *software* EchoPAC (GE).
