# Supplementary material for: Apresentação de Software para Pós-processamento de Curvas de Deformação Cardíaca: D-Station
Source: Arq Bras Cardiol. 2020 Apr 6;114(3):496–506. [Article in Portuguese] doi: 10.36660/abc.20180403 (PMC7792733; doi:10.36660/abc.20180403)
Supplement: Translation

# Translation: Software for Post-Processing Analysis of Strain Curves: The D-Station

**Authors:** Rafael Duarte de Sousa, Carlos Danilo Miranda Regis, Ittalo dos Santos Silva, Paulo Szewierenko, Renato de Aguiar Hortegal, Henry Abensur

**Affiliations:** 1 Brazil Instituto Federal de Educação, Ciência e Tecnologia da Paraíba, João Pessoa, PB – Brazil; 2 Brazil Instituto Dante Pazzanese de Cardiologia - Statistical Consultant, São Paulo, SP – Brazil; 3 Brazil Instituto Dante Pazzanese de Cardiologia, São Paulo, SP – Brazil; 4 Brazil Hospital Beneficência Portuguesa de São Paulo – Echocardiography Department, São Paulo, SP – Brazil

**Keywords:** Cardiovascular Diseases/diagnostic imaging, Prognosis, Echocardiography/methods, Ventricular Dysfunction, Left/physiopathology, Speckle Tracking

## Abstract

**Background:**

The use of speckle-tracking echocardiography for evaluation of cardiac function has great applicability in different scenarios. The broad use of this method requires tools that allow the extraction of relevant data from strain curves and inclusion of these data in traditionally used parameters.

**Objectives:**

The present study aimed to present and validate a free software, called D-station, for analysis of strain curves.

**Methods:**

From raw data files, the D-Station determines the phases of the cardiac cycle, and simultaneously exhibits the strain and strain rate curves of different cardiac chambers. Validation of the software was done by global longitudinal strain (GLS), and the analyses were performed: 1) graphical comparison of EchoPAC and D-Station paired measurements in relation to equality line; 2) by coefficient of correlation of these measurements; 3) test of hypothesis (p > 0.05); and 4) Bland-Altman analysis.

**Results:**

The Spearman’s rho correlation coefficient indicated a strong correlation between the measurements. Results of the test of hypothesis showed a p-value = 0.6798 >> 0.05, thus also indicating an equivalence between the softwares. The Bland-Altman analysis revealed a bias ≤ 1% and dispersion ≤ 2% between the measurements. The tests showed that, for GLS values lower than 10%, there was a trend for higher percentage difference between the values, although the absolute values remained low.

**Conclusion:**

The D-Station software was validated as an additional tool to the EchoPAC, which uses the raw data from the strain and strain rate curves exported from a proprietary software. (Arq Bras Cardiol. 2020; 114(3):496-506)

## Introduction

Analysis of cardiac strain by speckle tracking echocardiography has great applicability in different scenarios, including clinical cardiology practice^1^and research,^2^providing information about local and global mechanics of cardiac chambers.

Although left ventricular global longitudinal strain (GLS) is a robust parameter of cardiac function,^1 - 3^it assesses cardiac strain between the onset of isovolumetric contraction and the end of ventricular ejection. Therefore, valuable information of other phases, like isovolumetric relaxation, is not measured by the GLS.

Therefore, other tools are needed to obtain relevant data from the strain curve that can be used as additional methods to currently used ones.

Most of offline softwares supplied by different manufacturers (proprietary softwares) has preset analysis modes and parameters of cardiac strain. If on the one hand, this can make the software simpler and user-friendlier in daily clinical practice, on the other, makes it difficult to use this technology in research. In addition, the access to these tools may be limited and expensive.

International reference centers for study on cardiac strain usually have customized softwares that allow offline processing, without exclusive rights established by the manufacturers, and adjustments to their needs.^4^

The present study aims demonstrate the use of a new, free software called D-station, as an additional tool for the analysis of strain curves provided by any proprietary software. Besides, the study aims to validate this new software by comparison of its GLS values with GLS values obtained by the EchoPAC (GE) software.

## Methods

### D-Station: post-processing software for strain curve analysis

D-Station is a free, customized software written in Python 3, designed to enable an offline post-processing of the strain curves. The steps of execution of D-Station program are illustrated in Figure 1 . D-Station does not replace pre-existing platforms, but rather expands the possibilities of post-processing.

**Figure 1:** – D-Station algorithm. ECG: electrocardiogram.

### Separation into phases

Each strain curve corresponds to one or more cardiac cycles in certain region of the cardiac chamber and can be divided into the mechanical phases of this cycle. According to previous studies,^4^definition of these phases relies on the times of opening and closing of the aortic and mitral valves, on the time of electrical events, obtained from electrocardiogram (ECG) waves, as well as the time of the onset of the first and the second QRS complex, and onset of P-wave.^5 , 6^The ECG curves match well with the strain curve and the strain rate (SR) in the files.

Considering the onset of the cardiac cycle at the onset of the QRS complex, six phases were defined, as follow (in order of occurrence): electrical mechanical coupling (EMC), isovolumic contraction (IC), ejection phase (Ejec), isovolumic relaxation, early filling (E), atrial contraction (A). A detailed description of definitions of each phase of the cardiac cycle is provided in the supplementary material.

### Algorithm of reading of the signs and parameters calculation

The program entries are: 1) time of opening and closing of aortic and mitral valves; 2) raw data files containing the strain curves or strain rate; 3) identifier of the test; and 4) visualization option selected by the user. Further information can be found in the software manual, presented in the supplementary material of the study.

### Six visualization options are available in the current version of the software:

- *Strain - LV* (left ventricular strain), *strain rate - LV* (left ventricular SR) and ECG;
- *Strain - LV* , *strain - LA* (left atrial s *train* ) and ECG;
- *Strain - LV* , *strain rate - LA* and ECG;
- *Strain - LV* , *strain - RV* (right ventricular strain) and ECG;
- *Strain - LV* , *strain rate - LV* and ECG, where *SR* is obtained from the strain curves;
- *Test option (CircAdapt interface): strain - LV* and *strain rate - LV*

In all these options, curves are exhibited simultaneously as shown in Figure 2 .

**Figure 2:** – Left ventricular strain, left atrial strain, and echocardiographic curves with division into cardiac cycle phases. Eighteen strain curves corresponding to 18 segments of the left ventricle, six left atrial strain curves, and one electrocardiographic signal. Colors of the strain and strain rate curves correspond to those attributed to the segments by the proprietary software; MVC: mitral valve closure; AVO: aortic valve opening; AVC: aortic valve closure; MVO: mitral valve opening.

From raw data containing information of three-, four-, and two-chamber planes, left ventricular strain curves can be visualized, according to the model of the 18 segments proposed by the American Heart Association (AHA).^7^

Processing of the raw data sheets consists in changing the format to optimize the software functioning. In addition, due to small changes in heart rate on ECG curves, the four-chamber apical view was adopted as standard. After formatting of the sheets, a picture containing strain, SR and ECG curves is exhibited. The user should then define three points in the figure – the onset of QRS complex, the onset of P-wave and the onset of the second QRS complex.

Based on the values obtained form these points and timing of the opening and closing of the valves, it is possible to determine each phase of the cardiac cycle. The D-station terminal exhibits the time points of each of these phases, as well as the values of each calculated parameter. The user can decide between a picture containing the curves of cardiac chambers of interest ( Figure 3 ) or the picture containing the points used in the parameters’ calculation.

**Figure 3:** – Simultaneous visualization of strain longitudinal curves of the left (18 segments) and the right (six segments) on the right. Time of the onset of the phases and parameters calculated in the terminal on the left. Other configurations can be accessed through the options available; MVC: mitral valve closure; AVO: aortic valve opening; AVC: aortic valve closure; MVO: mitral valve opening.

### Event timing and calculated parameters

Each of the longitudinal strain curves presented in Figure 3 has an important event for the calculation of the software’s parameters: the peak systolic strain, defined as the peak value during systole, according to the EACVI/ASE.^7^

The peak systolic strain of each segment is used for calculation of GLS, defined as the arithmetic mean of peak systolic strain values of all segments.

All these possibilities of post-processing allow and/or facilitate the analysis of new parameters, including the strain/SR of left and right atrium, right ventricular strain and diastolic recovery (diastolic stunning)^8^for example.

### Algorithm for recognition of the peak systolic strain

The D-Station defines the peak systolic strain as the most negative strain value between the onset of the QRS and the AVC. This contrasts with the EchoPAC software, which determines the peak systolic strain according to the criterion presented in Figure 4 .

**Figure 4:** – EchoPAC selection criterion of the peak systolic strain.

### Validation of the D-Station: database and statistical analysis

To validate the D-Station software, files containing strain curves of 48 individuals were obtained from the database of the Division of Echocardiography of *Hospital Beneficiencia Portuguesa de São Paulo* . We did not perform a sample calculation, and hence a convenience sample was selected by retrospective analysis of the database. All tests were performed after participants signed an informed consent form. The study was approved by the Ethics Committee of the institution (CAEE approval number 91350318.4.0000.5483).

The time of opening and closing of the mitral and aortic valves were registered. Some test results showed more than one event time registered; tests with discrepancies of time higher than 10 ms were excluded.

The cardiac cycle with the best image quality in the apical three- four- and two-chamber view was selected. In case of three cycles with poor-quality image, the last cycle was selected. The endocardial board was defined by delineation of the region of interest using the option *Q-analysis* of the EchoPAC software. A visual inspection of the tracking quality was made, which was confirmed by the “approve” option, and finally the GLS_EchoPAC value was registered. In case of poor-quality tracking (by visual inspection), this process was repeated. Tests with two or more segments with suboptimal quality were excluded.

The raw data of the strain curves were extracted using the “Store Trace” option, which generates .txt files that are used in data processing in D-Station.

The GLS was chosen as a parameter of validation of measurement equivalence in the EchoPAC processing (a well-established technique – gold standard) and the D-station (the proposed technique), showed in Table 1 .

**Table 1:** – Global Longitudinal Strain (%) obtained by EchoPAC and D-Station

Subject	GLS_Echopac	GLS-D-Station	Subject	GLS_Echopac	GLS-D-Station	Subject	GLS_Echopac	GLS-D-Station
1	–17.90	–17.88	17	–19.00	–19.03	33	–24.40	–24.62
2	–7.90	–9.50	18	–16.90	–16.82	34	–19.10	–19.57
3	–10.50	–11.10	19	–19.500	–16.68	35	–7.40	–6.46
4	–8.50	–8.19	20	–19.80	–19.83	36	–2.70	–3.37
5	–13.30	–13.55	21	–16.70	–17.04	37	–5.70	–5.22
6	–18.40	–18.26	22	–20.50	–20.93	38	–4.50	–4.32
7	–4.60	–4.21	23	–14.90	–14.71	39	–10.50	–9.83
8	–21.60	–21.48	24	–20.20	–19.76	40	–9.40	–10.95
9	–16.20	–16.36	25	–17.80	–18.19	41	–10.60	–10.47
10	–11.90	–11.41	26	–20.10	–20.47	42	–11.10	–11.15
11	–8.80	–7.33	27	–17.30	–17.60	43	–3.20	–3.69
12	–17.30	–17.23	28	–17.50	–16.96	44	–8.20	–8.64
13	–20.40	–20.32	29	–21.20	–20.28	45	–6.60	–6.01
14	–19.80	–19.40	30	– 23.00	–23.06	46	–6.90	–6.85
15	–16.40	–15.27	31	– 20.70	–19.91	47	–10.60	–10.11
16	–19.20	–19.38	32	–21.10	–21.22	48	–8.80	–9.28

Methods used in the analyses:

- Normality test of GLS obtained by EchoPAC, D-Station and the differences (EchoPAC – D-Station), using a graphical method (Q-Q plot), followed by a statistical method (Shapiro-Wilk test) to confirm normality assumption found by the graphical method;
- Graphs of GLS by EchoPAC and D-Station in case of equality or coefficient of correlation (Pearson’s correlation or Spearman’s correlation for normal and non-normal distribution, respectively, of EchoPAC and D-Station data);
- Test of the hypothesis of difference between GLS values by EchoPAC and D-Station GLS, paired data, level of significance of 5% by Student’s t-test or the non-parametric Wilcoxon test in case of normal and non-normal distribution of data, respectively.
- Agreement test by Bland-Altman plot^9 , 10^

The Stats and the BlandAltmanLeh packages of the R software version 3.5.2 (2018-12-20) were used, which has the necessary commands and outputs for p-value calculation and Bland-Altman analysis.

### Validation criteria

From the clinical point of view, the criteria used to determine whether D-Station can be used as an alternative method to EchoPAC (equivalence), were the following:

- Normality test The analysis using the Q-Q plot is visual and hence subjective. If data are normally distributed, the points lie on a straight line constructed with data analyzed. The assumption of normality (Shapiro-Wilk test) was accepted if p-value was > α (level of significance = 5%).
- Spearman correlation coefficient ≥0.95.
- Hypothesis testing: p-value>0.05 (equivalence between the measurements) Ho: mean difference (EchoPAC - D-Station) = 0 Ha: mean difference ≠ 0
- Bland-Altman

- Systematic error (bias) ≤ 1%
- scattering of the data ≤ 2%

(*) Please note that the unit of measurement of GLS is % and therefore these values refer to absolute variation.

## Results

### Simultaneous visualization of the curves in different cardiac chambers

The D-Station software provides the simultaneous display of all strain curves and SR of different cardiac chambers, allowing the study of the interaction between them. Additional options including combinations of different displays can be easily added to the program, with consequent extraction of other parameters for the study on cardiac strain in different chambers simultaneously and by cardiac cycle. As example, exhibits the curves of left and right ventricles, which facilitates the analysis of the interactions between them.

### CircAdapt Interface: generation of virtual cardiac models

The D-Station “Test” option has been designed to define the strain curve parameters without separation into phases. Consequently, the ECG curve is no longer necessary, and the program becomes compatible with the mathematical model CircAdapt. This model, combined with the MultiPatch Module, proposed by Walmsley et al.,^11^can retrieve the strain curves corresponding to simulations and the times of mechanical events, without ECG signals, as shown in Figure 6 . Thus, the D-Station software can work with virtual cardiac models developed according to Walmsley et al.^11 - 14^

**Figure 6:** – Simultaneous display of 18 strain curves of the left ventricle, 18 strain rate curves of the left ventricle obtained by CircAdapt; thus, there is no electrocardiographic signal or separation into phases; MVC: mitral valve closure; AVO: aortic valve opening; AVC: aortic valve closure; MVO: mitral valve opening.

### Applicability of machine learning techniques

Machine learning consists of a subset of artificial intelligence, capable of processing complex problems of interaction between variables and making accurate predictions. It has been widely used in different areas of cardiology. The storage format of entries and data obtained by the program allows the implementation of machine learning algorithms and thereby the automatic extraction of parameters, classification of a large number of signals and reading of space-time characteristics of the entire strain curve, as proposed by Tabassian et al.^15^

### Validation analysis results

#### a) Normality testing of measures

Figure 7 shows the Q-Q plot of EchoPAC ( Figure 7a ), D-Station ( Figure 7b ) and EchoPAC - D-Station ( Figure 7c ). As can be seen in Figures 7a and 7b , several points are out of the red reference line, indicating that EchoPAC and D-Station data are not normally distributed. On the other hand, in Figura 7c , most of the points lie on or are very close to the red reference line (except for two points in the right upper corner), indicating that the difference between the measurements tend to be normally distributed.

**Figure 7:** – Q-Q plots.

Since the difference between measurements will be used in the hypothesis test, we sought to confirm the hypothesis of normality in the distribution of these differences obtained by the graphical method by using the Shapiro-Wilk test, which confirmed the hypothesis of normality (p > 0.05) ( Figure 8 ).

**Figure 8:** – Shapiro-Wilk normality test.

#### b) Graphs of EchoPAC and D-Station measurements in relation to equality line and coefficient of correlation

Figure 9 shows the distribution of EchoPAC and D-Station (paired data) in relation to the equality line, evidencing a distribution of points close to and in both sides of the line, suggesting a low bias from the qualitative viewpoint and scattering. Since these measures did not have a normal distribution, we used the Spearman correlation test, which indicated a strong correlation (r = 0.99) between results obtained by the two methods.

**Figure 9:** – Global longitudinal strain values obtained by EchoPAC and D-Station in relation to the equality line.

#### c) Hypothesis test of the differences between EchoPAC and D-Station GLS values

Since the differences between the measurements had normal distribution, we used the paired t-test (significance level of 5%). Results are presented in Figure 10 , with a p-value of 0.6798, indicating acceptance of null hypothesis, *i.e* ., equivalence between the methods.

**Figure 10:** – Paired t-test

#### d) Bland-Altman agreement analysis9,10

Figure 11 despicts the Bland-Altman plot, which indicates agreement between the two methods as they meet the third (c) validation criterion . There is an evidence of large % differences for absolute (module) values of GLS < 10%.

**Figure 11:** – Differences and means of global longitudinal strain (GLS) values obtained by EchoPAC and D-station.

## Discussion

### Analysis of agreement between the methods

Validation analysis results met the validation criteria, indicating equivalence between GLS values obtained by EchoPAC and D-Station. In a detailed analysis of the data, we can see that, for values lower than 10%, there was a trend of higher percentage difference. Intriguingly, all these subjects had important ventricular dysfunction with intraventricular dyssynchrony of left bundle branch block type. Such discrepancies may be precipitated by some factors, as follow:

1. Low absolute values result in higher percentage differences;
2. Ventricular dyssynchrony with left bundle branch block usually presents a stretching of the basal segment of the inferolateral and/or anterolateral wall at the beginning of systole, as well as erratic, mid- and telesystolic movements of the septum after the typical “septal flash”. Both can generate positive peaks. While D-Station defines systolic peak as the most negative value, regardless of the positive (or less positive) peak in case of exclusively positive curves, the EchoPAC assumes, as a rule for systolic peak (peak systolic strain), a positive peak 75% greater than the negative systolic peak mode value, as shown in Figure 4 . Also, in EchoPAC, although manual adjustments are common in these cases, we decided not to make these adjustments aiming at greater accuracy of the method.

In summary, discrepancies in the definition of systolic peak reduce the reproducibility of GLS between programs in patients with left bundle branch block. This issue should be addressed in future studies.

However, these discrepancies do not have a negative impact, especially if we consider the intraobserver variability of GLS values reported in the literature (5.2%),^16^and inter-software discrepancies regarding speckle filtering and tracking.^17 - 19^

Therefore, analysis of the results validates the D-Station as an alternative to EchoPAC.

### Potential Applications of the D-Station Software

There are numerous potential applications of the D-Station software: simultaneous analysis of different chambers allows the study on the interaction between left and right ventricles, as well as left ventricle and left atrium, which may be relevant in heart failure with preserved ejection fraction, pericardial disease and interventricular dyssynchrony.

The interface of D-Station with Circadapt model combined with the MultiPatch module allows the formulation of hypotheses and comparison of signals between real patients, as previously performed.^12 - 14^This contributes with the teaching of the pathophysiology of cardiac strain, in addition to potentially reduces the time to select the variables of interest and spare resources in the development of animal models in some research scenarios.

The machine learning technique may be configured to process a great number of signals, identify variables of interest by data mining, and enable the use of the points of the strain curve/SR as described by Tabassian et al.^15^This can lead to extraction of further relevant data obtained from the study on cardiac strain, potentiated by the machine learning techniques, mainly by the imminent arrival of the high frame rate speckle tracking.^20^

Finally, future updates may expand the possibilities of analysis, including st *rain* radial, circumferential and Twist, as well as the optimization of the interface between proprietary softwares, by incorporating strain parameters, Doppler signals, chamber volumes, tissue Doppler, among others. This will allow the automated extraction of many new, pre-established parameters at the user’s discretion.

### Limitations

The current version of the D-Station software does not allow the update of visualizations. In other words, to alter the chamber selection and its strain/SR curves, the user must restart the program. The same occurs in case of erroneous definition of the points on the ECG curve.

Differences in the measurement of cardiac strain between manufacturers are a critical issue in speckle tracking, as previously discussed by Mirea et al.^18^Further studies are needed to evaluate the impact of this software on discrepancies between manufacturers.

## Conclusion

The D-Station software is an additional tool for the assessment of strain curves obtained by raw data exported from another proprietary software, with good correlation in the measurement of GLS as compared with the EchoPAC (GE) software.

**Figure 5:** – Simultaneous display of 18 strain curves of the left ventricle, six strain curves of the right ventricle and electrocardiographic curve; MVC: mitral valve closure; AVO: aortic valve opening; AVC: aortic valve closure; MVO: mitral valve opening.
